# The first 1000 symptomatic pediatric SARS-CoV-2 infections in an integrated health care system: a prospective cohort study

**DOI:** 10.1186/s12887-021-02863-1

**Published:** 2021-09-13

**Authors:** Leigh M. Howard, Kathryn Garguilo, Jessica Gillon, Kerry LeBlanc, Adam C. Seegmiller, Jonathan E. Schmitz, Daniel W. Byrne, Henry J. Domenico, Ryan P. Moore, Steven A. Webber, Natasha B. Halasa, Ritu Banerjee

**Affiliations:** 1 Department of Pediatrics, Vanderbilt University Medical Center, Nashville, TN USA; 2 Department of Pathology, Microbiology, and Immunology, Vanderbilt University Medical Center, Nashville, TN USA; 3 Department of Biostatistics, Vanderbilt University Medical Center, Nashville, TN USA; 4 Medical Center North, 1161 21st Avenue South, Nashville, Tennessee USA

## Abstract

**Background:**

The spectrum of illness and predictors of severity among children with SARS-CoV-2 infection are incompletely understood.

**Methods:**

Active surveillance was performed for SARS-CoV-2 by polymerase chain reaction among symptomatic pediatric patients in a quaternary care academic hospital laboratory beginning March 12, 2020. We obtained sociodemographic and clinical data 5 (+/-3) and 30 days after diagnosis via phone follow-up and medical record review. Logistic regression was used to assess predictors of hospitalization.

**Results:**

The first 1000 symptomatic pediatric patients were diagnosed in our institution between March 13, 2020 and September 28, 2020. Cough (52 %), headache (43 %), and sore throat (36 %) were the most common symptoms. Forty-one (4 %) were hospitalized; 8 required ICU admission, and 2 required mechanical ventilation (< 1 %). One patient developed multisystem inflammatory syndrome in children; one death was possibly associated with SARS-CoV-2 infection. Symptom resolution occurred by follow-up day 5 in 398/892 (45 %) patients and by day 30 in 443/471 (94 %) patients. Pre-existing medical condition (OR 7.7; 95 % CI 3.9–16.0), dyspnea (OR 6.8; 95 % CI 3.2–14.1), Black race or Hispanic ethnicity (OR 2.7; 95 % CI 1.3–5.5), and vomiting (OR 5.4; 95 % CI 1.2–20.6) were the strongest predictors of hospitalization. The model displayed excellent discriminative ability (AUC = 0.82, 95 % CI 0.76–0.88, Brier score = 0.03).

**Conclusions:**

In 1000 pediatric patients with systematic follow-up, most SARS-CoV-2 infections were mild, brief, and rarely required hospitalization. Pediatric predictors of hospitalization included comorbid conditions, Black race, Hispanic ethnicity, dyspnea and vomiting and were distinct from those reported among adults.

**Supplementary Information:**

The online version contains supplementary material available at 10.1186/s12887-021-02863-1.

## Background

Clinical features and outcomes associated with SARS-CoV-2 infection may differ by age [1–5]. The spectrum of illness associated with SARS-CoV-2 infection in children is broad, including asymptomatic viral detection, and features of coronavirus disease 2019 (COVID-19), typically characterized by respiratory symptoms, fever, and sometimes diarrhea, headache, and anosmia [3, 6, 7]. The severity of illness has been reported to range from mild, transient symptoms to illness requiring hospitalization, intensive respiratory support, and even rarely death in children. There are increasing reports of a severe inflammatory syndrome occurring in the weeks after SARS-CoV-2 infection or exposure in children [8, 9], now known as multisystem inflammatory syndrome in children (MIS-C) [9–14]. However, current understanding of the full spectrum of manifestations of SARS-CoV-2 infection in children is very limited because most existing reports are derived from cross-sectional assessments of children with more severe illnesses, such as hospitalized children [3, 15–17]. Less is known regarding the symptoms, illness duration, and predictors of severe illness among ambulatory children diagnosed in the community with SARS-CoV-2 infection. Our objectives were to report the sociodemographic and clinical characteristics, including duration of symptoms, and clinical outcomes of children and adolescents diagnosed with SARS-CoV-2 infection in a large, integrated health network affiliated with an urban academic medical center in the southern United States (U.S.) and to identify predictors of hospitalization in these children.

## Methods

### Study design and population

Active laboratory surveillance was performed for all symptomatic children and adolescents with a positive SARS-CoV-2 test by polymerase chain reaction (PCR) in the clinical laboratory of a large quaternary care academic medical center in the southeastern U.S. The pediatric network of care of this medical center includes a large acute care pediatric hospital with 343-beds, with over 16,000 inpatient discharges, 47,000 emergency department (ED) encounters, and 360,000 ambulatory clinic encounters each year. SARS-CoV-2 testing was offered in the inpatient, ED, and ambulatory settings, which includes a primary care network, 9 urgent care clinics that see pediatric patients, 6 pediatric-only urgent care clinics, 2 dedicated on-campus SARS-CoV-2 testing sites, and 14 urgent care clinics located within private retail pharmacies. This network serves a diverse patient population derived from across urban, suburban, and rural Tennessee, southern Kentucky, and northern Alabama. The study was reviewed and approved by the Institutional Review Board.

### SARS-CoV-2 testing

Nucleic acid amplification testing for SARS-CoV-2 was performed with a variety of methodologies with Emergency Use Authorization by the US Food and Drug Administration (with multiple platform-use dictated by supply limitations). These platforms included a modified Centers for Disease Control and Prevention (CDC) qRT-PCR assay, the Roche 6800, the Hologic Panther, the GenMark Eplex, the Diasorin Liaison, the Biofire FilmArray SARS-CoV-2 monoplex, the Cepheid GeneXpert, and the Abbott IDNOW. The CDC assay employed the N1 and N2 primer/probe sets for SARS-CoV-2 with amplification/detection on an Applied Biosystems QuantStudio 7 Flex. Specimens were nasopharyngeal or nasal swabs in viral transport media.

### Indications and locations for testing

SARS-CoV-2 PCR testing became available in our institution on March 12, 2020. Given limited testing capacity, initial testing criteria were restricted to symptomatic individuals with new fever and respiratory symptoms or those with contact with a SARS-CoV-2 infected individual. Routine screening of asymptomatic individuals prior to hospital admissions, chemotherapy initiation, stem cell or organ transplant, or procedures requiring anesthesia began on May 4, 2020. If asymptomatic individuals were identified as subsequently developing symptoms, they entered our symptomatic cohort.

### Study follow-up

For symptomatic patients, our team performed follow-up by phone to obtain baseline sociodemographic characteristics and ascertain data on exposures, comorbidities, clinical symptoms, and illness status. The phone followups were performed by a clinical nurse using a pre-specified list of questions and symptom check list. Early follow-up (day 5 +/-3 after laboratory diagnosis) was conducted for all patients, but later follow-up (30 days after diagnosis) was limited to a subset of patients due to insufficient staff in the face of rapidly rising cases at the end of the study period. We also reviewed electronic medical records (EMR), when available, to collect additional clinical and demographic information.

### Statistical analysis

We modeled the association of several potential risk factors of interest on hospital admission using multivariable logistic regression. These factors included sociodemographic variables such as age, gender, racial, and ethnic backgrounds, and clinical factors including weight, body mass index (BMI), and the clinical symptoms known before admission (presence of shortness of breath, vomiting, fever), and pre-existing conditions. With only 41 hospitalizations, we limited the model to 4 predictors to avoid overfitting. The predictive accuracy of the final model was evaluated using the c-statistic, Brier score, and calibration curve. Bootstrap model validation was used to test for overfitting. Daily and 7-day moving average test positivity frequencies were calculated and stratified by asymptomatic and symptomatic status. Statistical analyses were conducted using STATA/SE 14.2 (StataCorp LP, College Station, TX) and R (version 3.5.0, r-project.org).

## Results

The first 1000 symptomatic pediatric patients identified in our healthcare network were diagnosed between March 13 and September 28, 2020 and their characteristics are summarized in Table 1 and Supplementary Fig. 1. Notably, 65 % had a known close contact with SARS-CoV-2 infection. Approximately 18 % of patients had at least one comorbidity. 801 (80 %) positive tests were collected in outpatient settings, 197 (20 %) in the ED, and 2 (< 1 %) during hospital admission. The median duration of symptoms prior to SARS-CoV-2 testing was 2 days (IQR 1–3 days, *n* = 754).

**Table 1:** Sociodemographic and clinical features of symptomatic SARS-CoV-2 positive children and adolescents^a^ diagnosed between 3/12/2020 and 9/28/2020^b^ (*n* = 1000 unless otherwise specified)

Characteristic or clinical variable	No. (%)
Age (y), median, IQR	14.0 (7.2–17.3)
Range	0.04–22.8
Age groups	
<1 year	87 (9)
1–4 years	99 (10)
5–9 years	140 (14)
10–14 years	230 (23)
≥15 years	444 (44)
Gender, female	523 (52.3)
Race^c^	
White	789 (78.9)
Black	129 (12.9)
Asian	45 (4.5)
Multiracial	72 (7.2)
Other or Unknown	19 (1.9)
Hispanic ethnicity	203 (20.3)
Testing location	
Outpatient clinic or testing site	801 (80.1)
Emergency department	197 (19.7)
Hospital admission	2 (0.2)
Method of data collection	
Patient interview	237 (23.7 %)
Medical record review	839 (83.9 %)
SARS-CoV-2 exposure	
Known household contact with COVID-19	409 (40.9)
Known community contact with COVID-19	241 (24.1)
Days to return test results (mean ± SD)	1.1 ± 1.3
Median (range)	1.0 (0–17)
Any comorbidity	177 (17.7)
Chronic lung disease	77 (7.7)
Neurologic condition	33 (3.3)
Cardiovascular disease	27 (2.7)
Diabetes mellitus	10 (1.0)
Immunocompromising condition	9 (0.9)
Chronic renal disease	8 (0.8)
Other chronic condition	65 (6.5)
Body mass index, median (IQR), *n* = 572	20.3 (17.1–23.8)
Underweight (BMI < 18.5)	165 (28.8)
Normal weight (BMI 18.5–24.9)	280 (49.0)
Overweight (BMI 25-29.9)	75 (13.1)
Obese (BMI ≥ 30)	52 (9.1)
Symptom resolution at early follow-up (*n* = 892)	398 (44.6)
Symptom resolution at later follow-up (*n* = 471)	443 (94.0)
Outcomes	
Hospitalization	
Remained outpatient	959 (95.9)
Admitted to hospital	41 (4.1)
ICU admission	8 (0.8)
Mechanical ventilation	2 (0.2)
Prescribed remdesivir	9 (0.9)
Death^4^	1 (0.1)
Clinical symptoms	
Headache	424 (42.4)
Sore throat	361 (36.1)
Fever > 100.4 F (38.0 C)	337 (33.7)
Nausea or vomiting	147 (14.7)
Shortness of breath (dyspnea)	98 (9.8)
Abdominal pain	82 (8.2)
Vomiting	16 (1.6)
Hypoxia	12 (1.2)

^a^Cohort includes 995 individuals < = 18 years of age and 5 individuals 19 and older who received long-term primary and subspecialty care in the pediatric facility (age range 19–22)

^b^Includes 1 infant who developed symptoms and positive SARS-CoV-2 test at 5 weeks of age after being born to mother who was SARS-CoV-2 positive at time of delivery

^c^Total *n* > 1000 as several subjects reported association with more than one race category

^d^Cause of death not definitively associated with SARS-CoV-2 infection in patient with multiple comorbidities

### Clinical features of illness

**Fig. 1:**
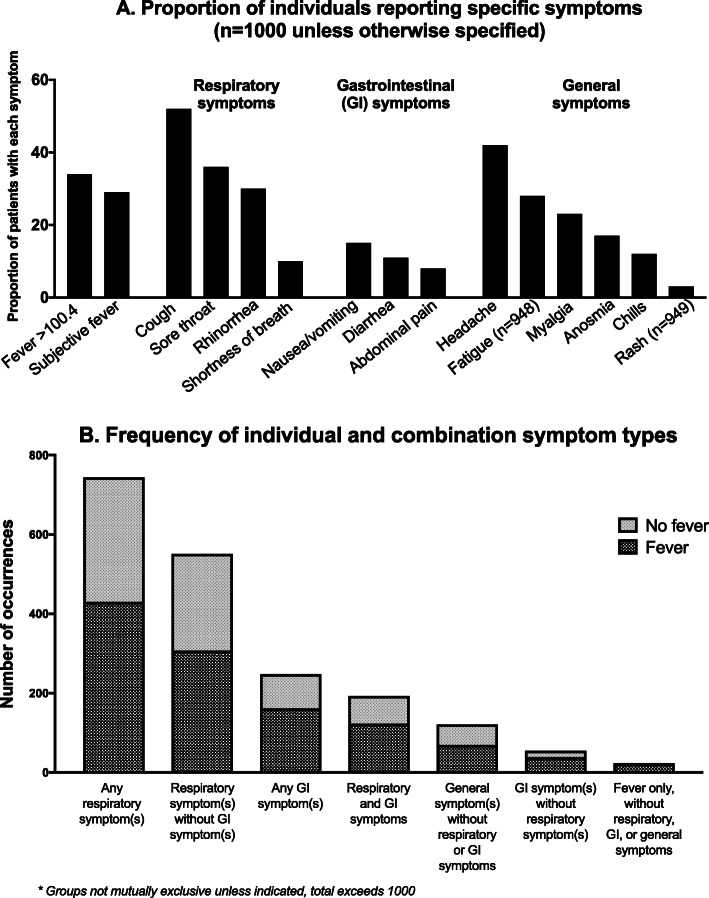
**A** Proportion of individuals reporting specific symptoms by symptom type (fever, respiratory symptoms, gastrointestinal symptoms, general symptoms); **B** Frequency of occurrence of symptom types (respiratory gastrointestinal, general) alone and in combination

Overall, the most common symptoms associated with SARS-CoV-2 infection were cough (52 %), headache (43 %), sore throat (36 %), and fever > 100.4 °F (34 %; Fig. 1, Supplementary Table 1). Symptom type and frequency varied according to age group, although some patient-reported symptoms, such as anosmia or headache, may have been more difficult to identify in younger children. Fever and rash were reported in a significantly higher proportion of younger children than older children and adolescents, while sore throat and myalgia were more frequent in the older age groups.

We classified symptoms as respiratory (cough, rhinorrhea, sore throat, shortness of breath), gastrointestinal (GI; nausea/vomiting, diarrhea, abdominal pain), or general (headache, myalgia, anosmia, fatigue, chills, rash). Overall, three-quarters (75 %) of patients exhibited at least one respiratory symptom, 66 % exhibited a general symptom, and 25 % had at least one GI symptom. Twenty-three (2 %) patients had fever alone without other symptoms, which occurred in a similar distribution across age groups (data not shown).

Of 744 patients with at least one respiratory symptom, 499 (67 %) also exhibited at least one general symptom and 193 (26 %) exhibited at least one GI symptom. General symptoms occurred without respiratory or GI symptoms in 121 (12 %) patients. Only 18 patients (2 %) had a GI symptom without a respiratory or general symptom present; 13/18 (72 %) of these patients’ GI symptoms were accompanied by documented or subjective fever.

### Hospitalizations

Forty-one (4 %) patients were hospitalized for possible or probable COVID-19 shortly after diagnosis of SARS-CoV-2 infection (Supplementary Table 2). Twenty-nine (71 %) of 41 hospitalized patients reported white race, while 24 % reported Black race, and 15 (37 %) reported Hispanic ethnicity (Table 2). Twenty-seven (66 %) hospitalized patients had at least one comorbidity. Eight patients (< 1 %) were admitted to the intensive care unit and two patients (< 1 %) required mechanical ventilation. One patient in our cohort later developed MIS-C. There was one death that was possibly related to SARS-CoV-2.

**Table 2:** Univariate comparison of patients not hospitalized vs. hospitalized among symptomatic SARS-CoV-2 positive children and adolescents diagnosed between 3/12/2020 and 9/28/2020

	Not hospitalized (*n* = 959)	Hospitalized (*n* = 41)	OR (95 % CI)	*p*-value
Age, mean ± SD, median (IQR)	12.1 ± 6.0, 14.1 (7.4–17.3)	10.0 ± 7.7, 12.2 (0.9–17.1)	0.95 (0.90-1.00)	0.077^b^
Male gender	459 (47.9 %)	18 (43.9 %)	0.9 (0.5–1.6)	0.619^a^
Race				0.086^a^
White	760 (79.2 %)	29 (70.7 %)	Reference	
Black	118 (12.3 %)	10 (24.4 %)	2.2 (1.1–4.7)	
Asian	41 (4.3 %)	2 (4.9 %)	1.3 (0.3–5.5)	
Other	40 (4.2 %)	0 (0.0 %)	0.0 (0.0-∞)	
Hispanic	188 (19.6 %)	15 (36.6 %)	2.4 (1.2–4.6)	0.008^a^
Black or Hispanic	304 (31.7 %)	25 (61.0 %)	3.4 (1.8–6.4)	< 0.001^a^
Body mass index, mean ± SD, median, BMI Category	21.9 ± 5.5, 21.0 (18.0-24.4)	22.5 ± 7.7, 20.9 (16.1–25.6)	1.02 (0.96–1.08)	0.849^b^ 0.435^a^
Underweight	153 (28.4 %)	12 (35.3 %)	1.49 (0.67–3.3)	
Normal weight	266 (49.4 %)	14 (41.2 %)	Reference	
Overweight	72 (13.4 %)	3 (8.8 %)	0.79 (0.22–2.83)	
Obese	47 (8.7 %)	5 (14.7 %)	2.02 (0.70–5.88)	
**Clinical Symptoms**				0.229^a^
Anosmia (loss of smell)	162 (16.9 %)	4 (9.8 %)	0.5 (0.2–1.5)	
Shortness of breath(dyspnea)	81 (8.4 %)	17 (41.5 %)	7.7 (4.0-14.9)	< 0.001^a^
Sore throat	357 (37.2 %)	4 (9.8 %)	0.2 (0.1–0.5)	< 0.001^a^
Vomiting	12 (1.3 %)	4 (9.8 %)	8.5 (2.6–27.7)	< 0.001^a^
Fever > 100.4 F	314 (32.7 %)	23 (56.1 %)	2.6 (1.4–4.9)	0.002^a^
Headache	420 (43.8 %)	4 (9.8 %)	0.2 (0.05–0.39)	< 0.001^a^
Hypoxia	1 (0.1 %)	11 (26.8 %)	351.3 (43.9-2809.2)	< 0.001^a^
Abdominal pain	74 (7.7 %)	8 (19.5 %)	2.9 (1.3–6.5)	0.007^a^
Nausea or vomiting	139 (14.5 %)	8 (19.5 %)	1.4 (0.6–3.2)	0.374 ^a^
**Pre-existing condition(s**)	150 (15.6 %)	27 (65.9 %)	10.4 (5.3–20.3)	< 0.001^a^
Immunocompromising condition	4 (0.4 %)	5 (12.2 %)	33.2 (8.5-128.7)	< 0.001^a^
Neurologic condition	24 (2.5 %)	9 (22.0 %)	11.0 (4.7–25.5)	< 0.001^a^
Cardiovascular disease	19 (2.0 %)	8 (19.5 %)	12.0 (4.9–29.4)	< 0.001^a^
Chronic renal disease	5 (0.5 %)	3 (7.3 %)	15.1 (3.5–65.4)	< 0.001^a^
Chronic lung disease	69 (7.2 %)	8 (19.5 %)	3.1 (1.4-7.0)	< 0.004^a^
Diabetes	9 (0.9 %)	1 (2.4 %)	2.6 (0.3–21.3)	< 0.344^a^
Other chronic condition	53 (5.5 %)	11 (26.8 %)	6.3 (3.0-13.2)	< 0.001^a^

^a^denotes a *P* value based on a chi-square test

^b^denotes a *P* value based a Mann-Whitney U test

^c^denotes a *P* value based on a Fisher’s exact test

### Symptom duration

Of 1000 patients, 892 (89 %) were reached for at least one early follow-up phone call (5 +/- 3 days after diagnosis). The median time of the early follow-up call was 2 days (IQR 2–3) after COVID-19 diagnosis. Complete symptom resolution occurred by the time of the early follow-up phone call in 398/892 (45 %) patients. The median age was greater among patients with ongoing symptoms vs. those with symptom resolution at the time of the early follow-up (14.8 years, IQR 7.4–17.4 vs. 12.4 years, IQR 6.0-16.6; Wilcoxon ranksum *p* = 0.001).

Symptom resolution occurred by day 30 in 443/471 (94 %) patients who could be reached for follow-up. Among 28 patients with ongoing symptoms at day 30, the median age was 13.3 years (IQR 3.9–16.8), and the most commonly reported ongoing symptoms were fatigue (6 patients), cough (5 patients), fever, and reduced sense of smell (4 patients each). No child was diagnosed with recurrent or “long” SARS-CoV-2 infection during the study period.

### Risk factors associated with COVID-19 hospitalization

**Fig. 2:**
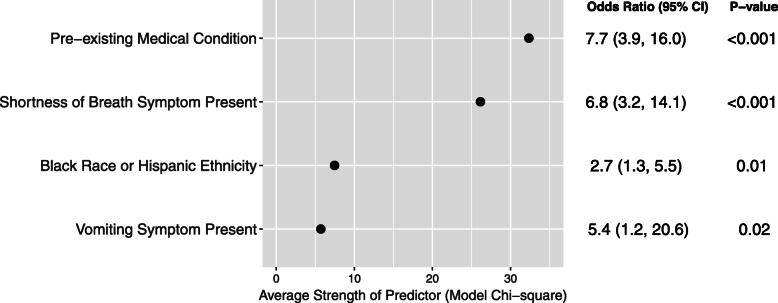
Visual representation of final multivariable logistic regression model predicting risk of hospitalization due to COVID-19. Scores are ordered by adjusted strength of each predictor as quantified by model chi-square statistic. The table also includes the odds ratio for each predictor, along with associated 95 % CI and *p*-values

We developed a logistic regression model to assess risk of hospitalization associated with COVID-19 (Fig. 2, Supplementary Table 3). The presence of at least one pre-existing medical condition (OR 7.7; 95 % CI 3.9 to 16.0), shortness of breath (OR 6.8; 95 % CI 3.2 to 14.1), Black race or Hispanic ethnicity (OR 2.7; 95 % CI 1.3 to 5.5), and vomiting (OR 5.4; 95 % CI 1.2 to 20.6) were the strongest predictors of hospitalization (Fig. 2). A tree diagram reporting the relative contributions of each identified predictor is presented in Supplementary Fig. 2. Within pre-existing conditions, immunologic, neurologic, and renal conditions were the strongest predictors of hospitalization (Supplementary Fig. 3). Body mass index (BMI) and weight were not significantly associated with hospitalization (Supplementary Tables 3, Supplementary Fig. 4). A predictive model incorporating these variables had an AUC of 0.82 (95 % CI 0.76 to 0.88) and Brier score of 0.03. The bootstrap model calibration using 40 replications showed minimal optimism in our estimates of model R-squared (0.28 apparent, 0.27 bias corrected), calibration intercept (0.0 apparent, -0.05 bias corrected), calibration slope (1.0 apparent, 0.98 bias corrected), and Brier score (0.03 apparent, 0.03 bias corrected) (Supplementary Fig. 5).

## Discussion

In this large prospective cohort study of the first 1000 children with SARS-CoV-2 infection in our health system, we conducted intensive phone followup and were able to characterize spectrum and duration of symptoms as well as identify novel predictors of hospitalization in the minority of children who required inpatient care. We found that most children with SARS-CoV-2 had good outcomes, remained outpatients, and nearly half had complete symptom resolution within 2 days of diagnosis. Our finding that pediatric illnesses were generally brief and mild, with only a small minority requiring hospital admission, intensive care, or mechanical ventilation are consistent with prior reports that children with SARS-CoV-2 infection experience less severe clinical outcomes than adults [1, 3, 6, 7, 16, 17].

The clinical features of illness in our cohort were similar to other pediatric studies from the U.S. and U.K, in which fever, cough, or shortness of breath occurred in approximately 75 % of pediatric patients [3, 7, 17, 18]. We found a slightly higher frequency of general symptoms such as headache, fatigue, and anosmia than in prior pediatric reports [3, 7]. Why children have milder COVID-19 phenotypes than adults is not well-established, although studies have suggested that risk of developing clinical symptoms following SARS-CoV-2 infection increases with age [19]. A recent study reported high colocalization of SARS-CoV-2 in lung cells expressing *TMPRSS2*, in which expression increases with age, suggesting a plausible explanation for age-related differences in SARS-CoV-2 susceptibility [20]. While children may have a lower susceptibility to SARS-CoV-2 infection and severe disease, nearly 3 million U.S. children have been infected to date, suggesting that the burden of pediatric disease is substantial [21]. A recent report of children in North Carolina identified Hispanic ethnicity and having a SARS-CoV-2 infected sibling as risk factors associated with SARS-CoV-2 infection after exposure [22]. The implications of these findings on public health measures to mitigate transmission are still being unraveled. While children are considered key drivers of transmission of many respiratory viruses, early data seemed to suggested that children may not be the primary drivers of SARS-CoV-2 transmission within households and communities [23]. This recent study from North Carolina and another household study from Tennessee and Wisconsin provide increasing evidence to support the potential role for children in SARS-CoV-2 transmission [24]. However, the efficiency of transmission in the setting of mild or subclinical infection is unclear. Thus, impact of community interventions to mitigate SARS-CoV-2 transmission aimed at children, such as school closures, may have relatively minor impacts compared with interventions that target older populations.

Our data suggest that predictors of hospitalization following COVID-19 diagnosis differ between children and adults. In adults, increasing age, male sex, and obesity have been reported as predictors of severe disease and poor outcomes associated with COVID-19 [25–27]. In our study, COVID-19 associated hospitalization in children was associated with comorbid conditions, Black race, Hispanic ethnicity, dyspnea and vomiting, but not age, BMI, weight, or male gender. Our findings support a recent assessment of > 5000 pediatric patients with SARS-CoV2 infection, in which an underlying medical condition and Black race were significantly associated with severe COVID-19 [28]. Our study, like many others, calls attention to the racial and ethnic disparities in SARS-CoV-2 infection rates and outcomes [22]. Associations between hospitalizations for COVID-19 and socioeconomic factors, social deprivation and access to healthcare warrant further study.

Our study has several strengths, including a large sample size and identification of all children with SARS-CoV-2 detections in an integrated health network including diverse ambulatory and inpatient settings, enabling a comprehensive assessment of the full spectrum of pediatric infections in the community. By prospectively following these subjects, we were able to assess not only the symptoms present at diagnosis, but also to follow the duration of symptoms.

Our study is also subject to some limitations. Our cohort was limited to patients who visited one of the testing sites. We had few patients who were hospitalized and hospitalization may not be a reliable surrogate for severe COVID-19 in children, as children with pre-existing conditions are more likely to be hospitalized for milder symptoms in the setting of the diagnosis of SARS-CoV-2 infection. Additionally, BMI information was unavailable in the EMR for many subjects.

Despite these limitations, we determined that while SARS-CoV-2 symptoms are generally mild and short-lived in most children, having a pre-existing medical condition is strongly associated with pediatric hospitalization following COVID-19 diagnosis. These findings can be used by clinicians and policy makers to prioritize at-risk pediatric populations for vaccination, novel therapeutics, and closer follow-up as the COVID-19 pandemic continues.

## Supplementary Information

**Additional file 1: Supplementary Table 1.** Clinical features of symptomatic SARS-CoV-2 infections among 1000 pediatric patients overall and by age group. **Supplementary Table 2.** Clinical characteristics of hospitalizations among the first 1000 symptomatic pediatric SARS-CoV-2 infections in our network (*n* = 41 patients hospitalized). **Supplementary Table 3.** Predictive Model of Hospitalization. **Supplementary Figure 1.** Histogram presenting age distribution (years) among 1000 symptomatic SARS-CoV-2 positive children and adolescents diagnosed between 3/12/2020 and 9/28/2020. **Supplementary Figure 2.** Tree diagram of the factors in the model for hospitalization. This classification and regression tree illustrated how the risk factors are related to hospitalization. Among the 1000 children with COVID-19, 4.1% (41) were hospitalized (1), 95.9% were not hospitalized. The strongest predictor of hospitalization was presence of a pre-existing condition (PEC). Among the 177 patients with a PEC, 15.3% were hospitalized vs. 1.7% of those without a PEC. Among those with a PEC, the next best predictor of hospitalization was shortness of breath (SOB). The hospitalization rate was 53.5% among those with both a PEC and SOB vs. 8.1% among those with a PEC but no SOB. Patients who are black or Hispanic were at increased risk of hospitalization among both those with and without PEC. **Supplementary Figure 3.** Tree diagram of ‘pre-existing conditions’ in the model for hospitalization, and the relative contributions of immunologic, neurologic, and renal conditions to the predictive model. **Supplementary Figure 4.** Association between Age, Weight, and Hospitalization. The locally weighted scatter plot smoothing lines illustrate the relationship between weight and age in the hospitalized and non-hospitalized patients. The dark bands represent the 95% confidence interval of each line. **Supplementary Figure 5.** Calibration curve for multivariate model predicting hospitalization. Calibration plot showing predicted probability of hospitalization vs. observed rate of hospitalization

## Data Availability

The datasets used and/or analysed during the current study are available from the corresponding author on reasonable request.

## Abbreviations

SARS-CoV-2: Severe acute respiratory syndrome coronavirus 2
COVID-19: Coronavirus disease 2019
MIS-C: Multisystem inflammatory syndrome in children
U.S.: United States
PCR: Polymerase chain reaction
VUMC: Vanderbilt University Medical Center
ED: Emergency department
CDC: Centers for Disease Control and Prevention
EMR: Electronic medical record
BMI: Body mass index
IQR: Interquartile range
GI: Gastrointestinal
