# Supplementary material for: The first 1000 symptomatic pediatric SARS-CoV-2 infections in an integrated health care system: a prospective cohort study
Source: BMC Pediatr. 2021 Sep 13;21:403. doi: 10.1186/s12887-021-02863-1 (PMC8435399; doi:10.1186/s12887-021-02863-1)
Supplement: Supplementary file 1 — Additional file 1: Supplementary Table 1. Clinical features of symptomatic SARS-CoV-2 infections among 1000 pediatric patients overall and by age group. Supplementary Table 2. Clinical characteristics of hospitalizations among the first 1000 symptomatic pediatric SARS-CoV-2 infections in our network (n = 41 patients hospitalized). Supplementary Table 3. Predictive Model of Hospitalization. Supplementary Figure 1. Histogram presenting age distribution (years) among 1000 symptomatic SARS-CoV-2 positive children and adolescents diagnosed between 3/12/2020 and 9/28/2020. Supplementary Figure 2. Tree diagram of the factors in the model for hospitalization. This classification and regression tree illustrated how the risk factors are related to hospitalization. Among the 1000 children with COVID-19, 4.1% (41) were hospitalized (1), 95.9% were not hospitalized. The strongest predictor of hospitalization was presence of a pre-existing condition (PEC). Among the 177 patients with a PEC, 15.3% were hospitalized vs. 1.7% of those without a PEC. Among those with a PEC, the next best predictor of hospitalization was shortness of breath (SOB). The hospitalization rate was 53.5% among those with both a PEC and SOB vs. 8.1% among those with a PEC but no SOB. Patients who are black or Hispanic were at increased risk of hospitalization among both those with and without PEC. Supplementary Figure 3. Tree diagram of ‘pre-existing conditions’ in the model for hospitalization, and the relative contributions of immunologic, neurologic, and renal conditions to the predictive model. Supplementary Figure 4. Association between Age, Weight, and Hospitalization. The locally weighted scatter plot smoothing lines illustrate the relationship between weight and age in the hospitalized and non-hospitalized patients. The dark bands represent the 95% confidence interval of each line. Supplementary Figure 5. Calibration curve for multivariate model predicting hospitalization. Calibration plot showing pr [file 12887_2021_2863_MOESM1_ESM.docx]

**The First 1000 Symptomatic Pediatric SARS-CoV-2 Infections in an Integrated Health Care System: A Prospective Cohort Study**

Leigh M. Howard, MD, MPH^1^ Kathryn Garguilo, RN, MSN^1^ Jessica Gillon, PharmD, BCPS^1^ Kerry LeBlanc, MSN, CPNP-PC^1^ Adam C. Seegmiller, MD, PhD^2^ Jonathan E. Schmitz, PhD, MD^2^ Daniel W. Byrne, MS^3^ Henry J. Domenico, MS^3^ Ryan P. Moore, MS^3^ Steven A. Webber, MBChB, MRCP^1^ Natasha B. Halasa, MD, MPH^1^ Ritu Banerjee, MD, PhD^1^

1. Department of Pediatrics, Vanderbilt University Medical Center, Nashville, TN
2. Department of Pathology, Microbiology, and Immunology, Vanderbilt University Medical Center, Nashville, TN
3. Department of Biostatistics, Vanderbilt University Medical Center, Nashville, TN.

**Supplementary Material**

| **Supplementary Table 1. Clinical features of symptomatic SARS-CoV-2 infections among 1000 pediatric patients overall and by age group** | | | | | | | |
| --- | --- | --- | --- | --- | --- | --- | --- |
| **Clinical feature** | **Age group** | | | | | | **p-value*** |
|  | **<1y**  **(n=87)** | **1-4y**  **(n=99)** | **5-9y**  **(n=140)** | **10-14y**  **(n=230)** | **15-18y**  **(n=444)** | **All ages**  **(n=1000)** | **Pearson chi-square/**  **chi-square for trend** |
| Fever >100.4 | 67 (77.0) | 51 (51.5) | 53 (37.9) | 63 (27.4) | 103 (23.2) | 337 (33.7) | <0.001/<0.001 |
| Subjective fever | 20 (23.0) | 28 (28.3) | 46 (32.9) | 69 (30.0) | 131 (29.5) | 294 (29.4) | 0.621/0.443 |
|  |  |  |  |  |  |  |  |
| Respiratory symptom (any) | 56 (64.4) | 63 (63.6) | 98 (70.0) | 169 (73.5) | 358 (80.6) | 744 (74.7) | <0.001/<0.001 |
| Cough | 36 (41.3) | 43 (43.9) | 69 (49.3) | 114 (49.6) | 254 (57.2) | 516 (51.6) | 0.014/.001 |
| Rhinorrhea | 38 (43.7) | 45 (45.5) | 38 (27.1) | 54 (23.5) | 123 (28.3) | 298 (29.8) | <0.001/<0.001 |
| Sore throat | 1 (1.2) | 10 (10.1) | 39 (27.9) | 97 (42.1) | 214 (48.2) | 361 (36.1) | <0.001/<0.001 |
| Shortness of breath | 8 (9.2) | 4 (4.0) | 5 (3.6) | 18 (7.8) | 63 (14.2) | 98 (9.8) | <0.001/0.001 |
|  |  |  |  |  |  |  |  |
| Gastrointestinal symptom (any) | 23 (26.4) | 20 (20.2) | 43 (30.7) | 64 (27.8) | 98 (22.1) | 248 (24.8) | 0.148/0.388 |
| Nausea or vomiting | 8 (9.2) | 10 (10.1) | 22 (15.7) | 42 (18.3) | 65 (14.6) | 147 (14.7) | 0.184/0.140 |
| Diarrhea | 18 (20.7) | 10 (10.1) | 13 (9.3) | 19 (8.3) | 48 (10.8) | 108 (10.8) | 0.029/0.086 |
| Abdominal pain | 0 (0) | 6 (6.1) | 23 (16.4) | 25 (10.9) | 28 (6.3) | 82 (8.2) | <0.001/0.618 |
| Vomiting^+^ | 2 (12.5) | 2 (8.3) | 3 (6.4) | 0 (0) | 9 (5.6) | 16 (4.8) | 0.028/0.283 |
|  |  |  |  |  |  |  |  |
| General symptom (any) | 23 (26.4) | 33 (33.3) | 76 (54.3) | 172 (74.8) | 353 (79.5) | 657 (65.7) | <0.001/<0.001 |
| Headache | 1 (1.1) | 7 (7.1) | 55 (39.2) | 111 (48.3) | 250 (56.3) | 424 (42.4) | <0.001/<0.001 |
| Fatigue (n=948) | 14 (16.9) | 14 (14.4) | 28 (21.5) | 64 (28.8) | 144 (34.6) | 264 (27.8) | <0.001/<0.001 |
| Myalgia | 2 (2.3) | 2 (2.0) | 23 (16.4) | 54 (23.8) | 147 (33.1) | 228 (22.8) | <0.001/<0.001 |
| Anosmia | 0 (0) | 1 (1.0) | 7 (0.05) | 40 (17.3) | 118 (26.6) | 166 (16.6) | <0.001/<0.001 |
| Chills | 0 (0) | 6 (6.1) | 15 (10.7) | 19 (8.3) | 78 (17.6) | 118 (11.8) | <0.001/<0.001 |
| Rash (n=949) | 9 (10.8) | 9 (9.3) | 0 (0) | 2 (0.9) | 4 (1.0) | 24 (2.5) | <0.001/<0.001 |
| ^+^ Vomiting: total n=332 (n=16 <1y, n=24 1-4y, n=47 5-9y, n=84 10-14y, n=161 ≥15 y) | | | | | | | |

**(Supplementary Table 1, continued)**

The following spline graphs represent the information in Supplementary Table 1. The blue lines represent the patients not hospitalized. The red lines represent the 41 patients hospitalized. The y-axis represents the proportion with each clinical feature by age. The number of patients, or data density, is marked by vertical lines. The clinical features most strongly associated with age were: headache, fever (highest in young patients), anosmia, sore throat, and myalgia. Sore throat was more common in those not hospitalized.


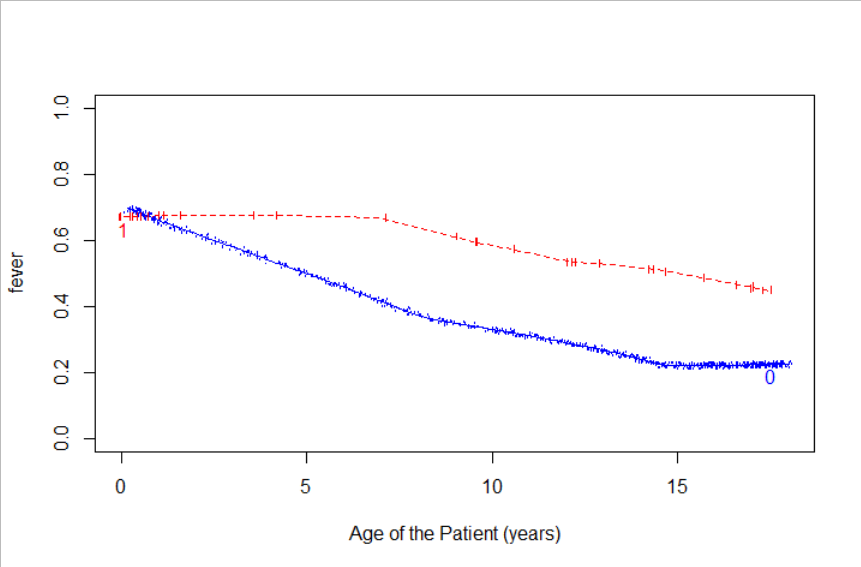

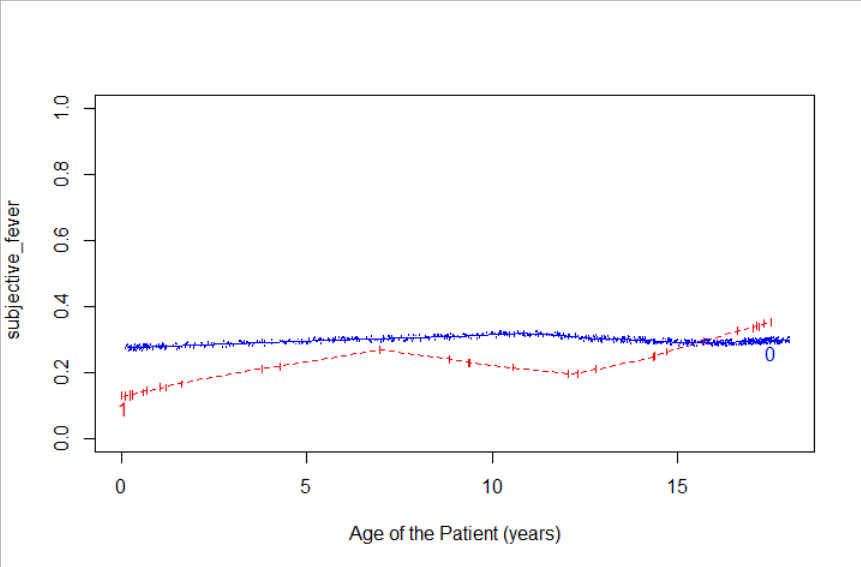


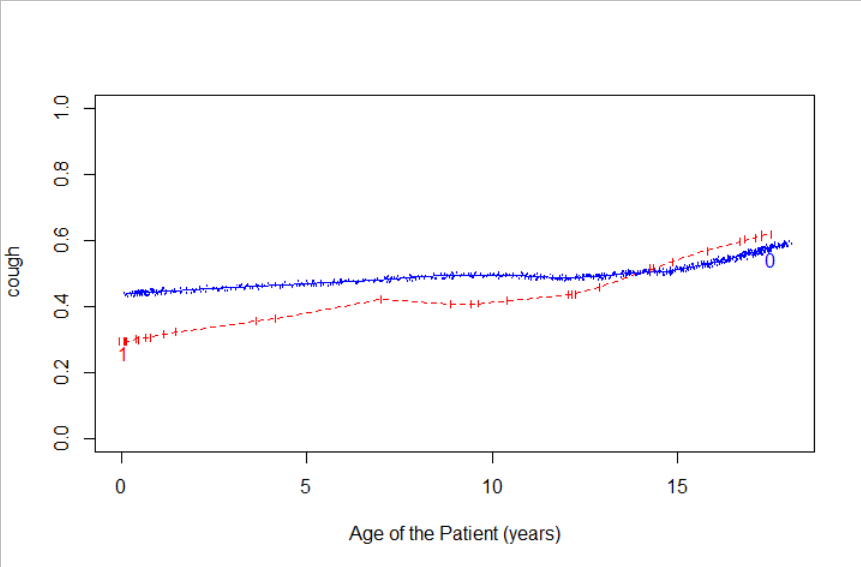

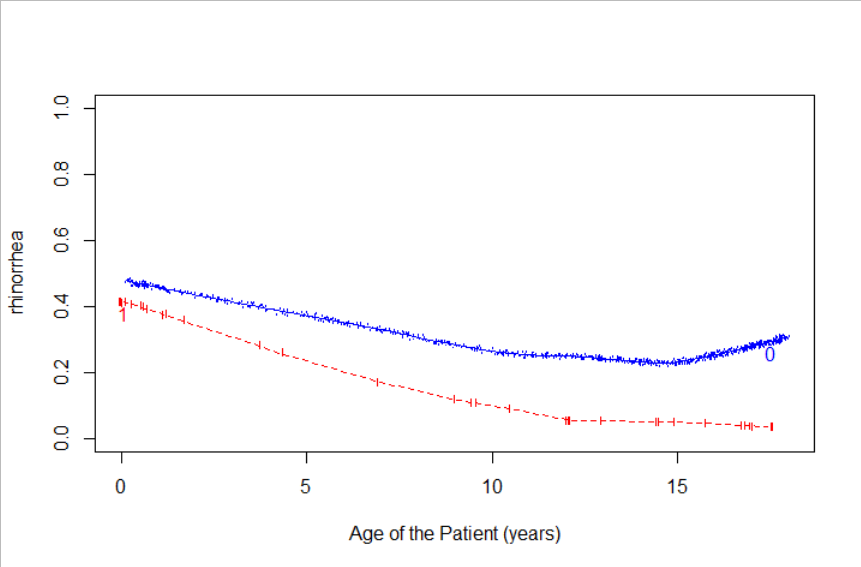


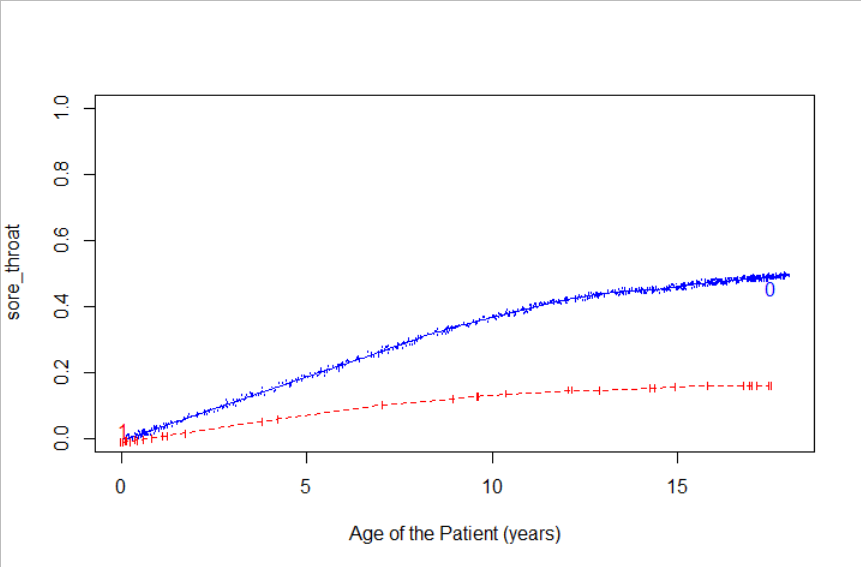

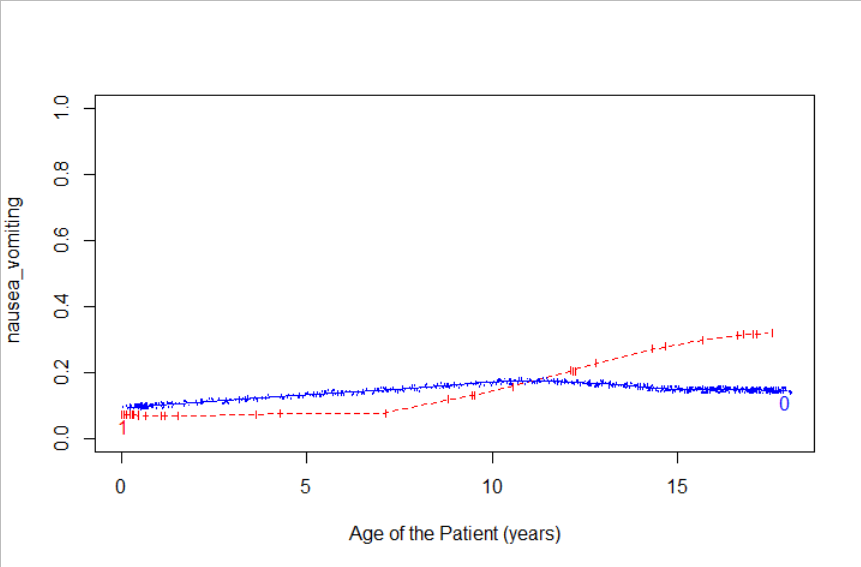

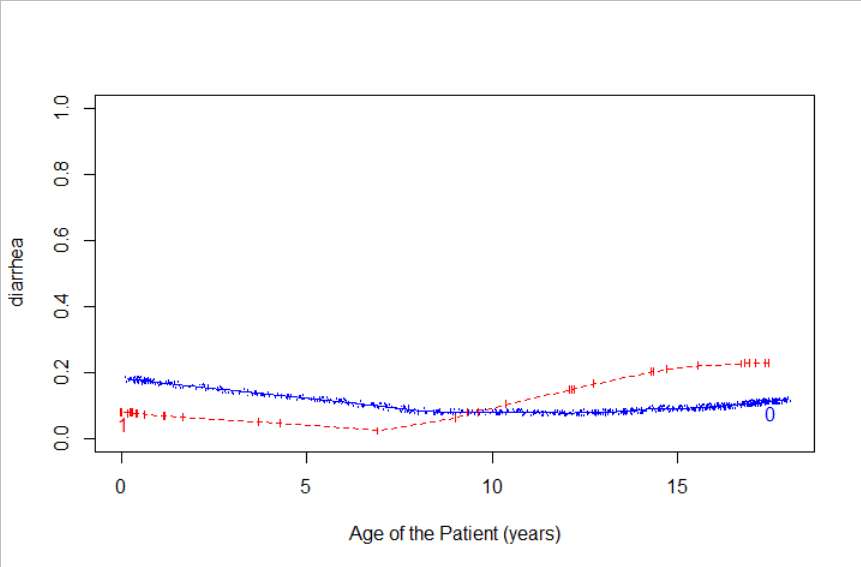

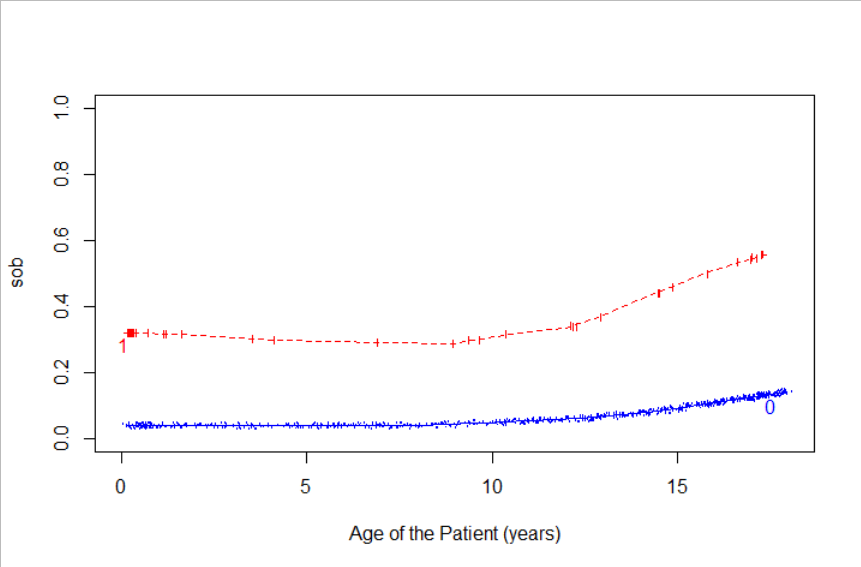


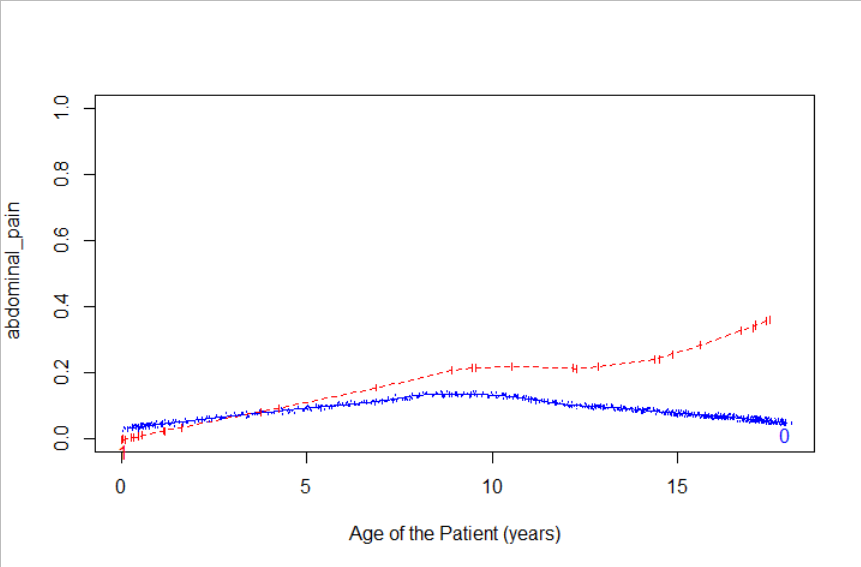

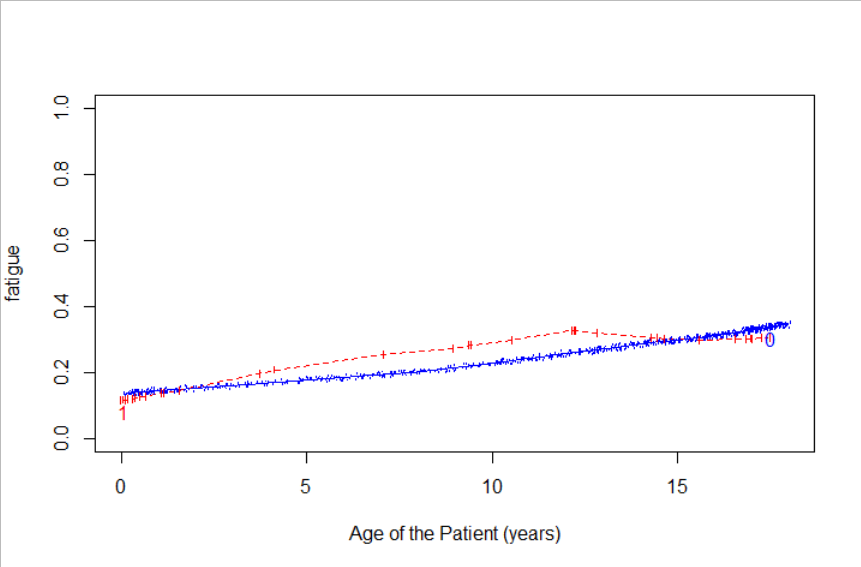


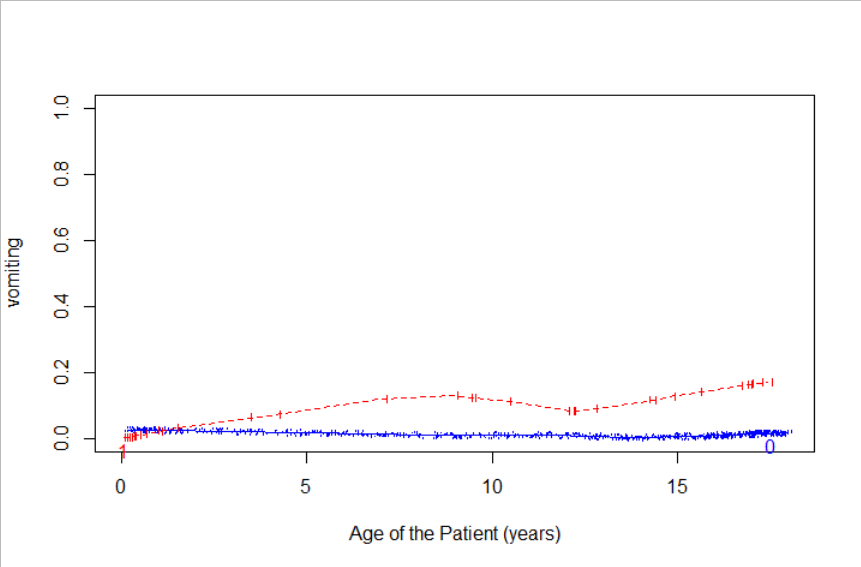

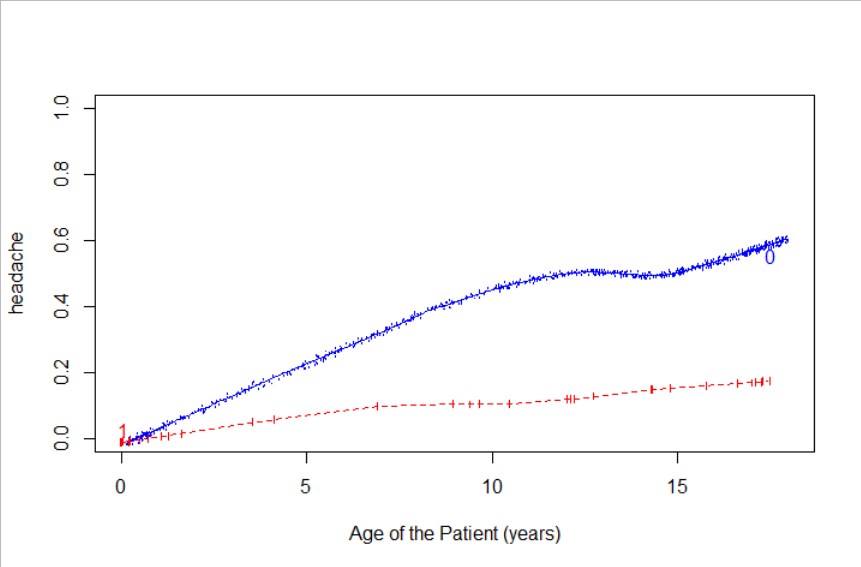


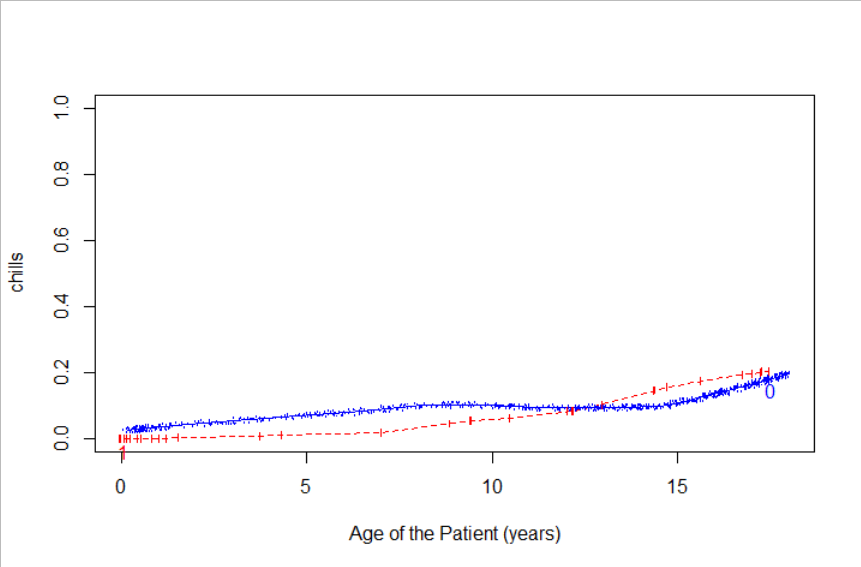

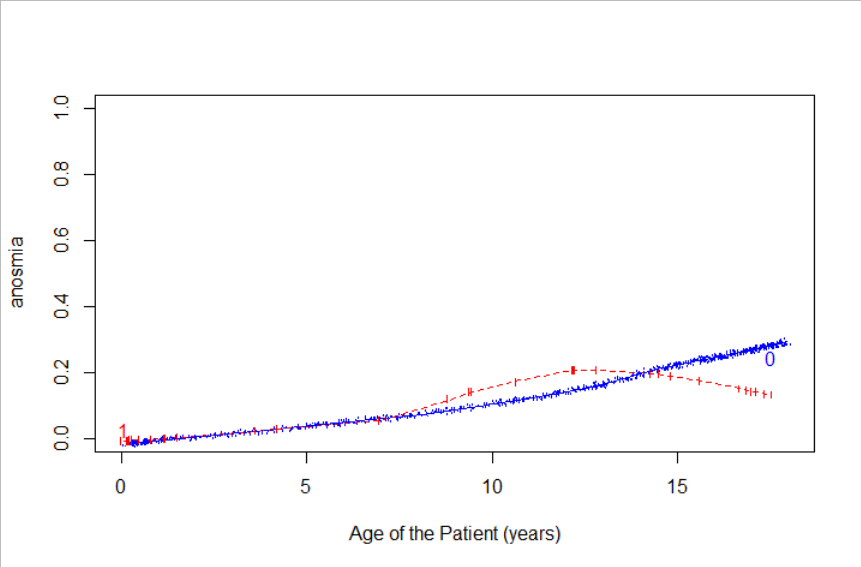

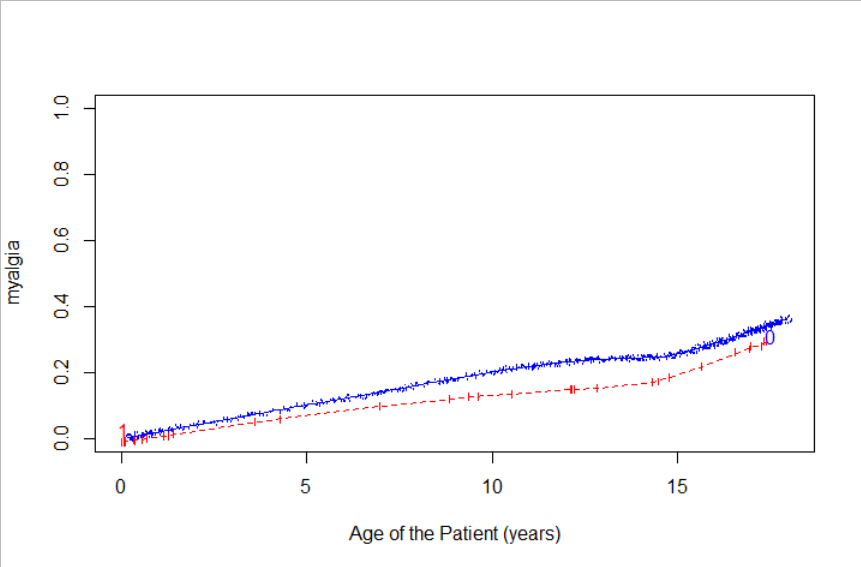

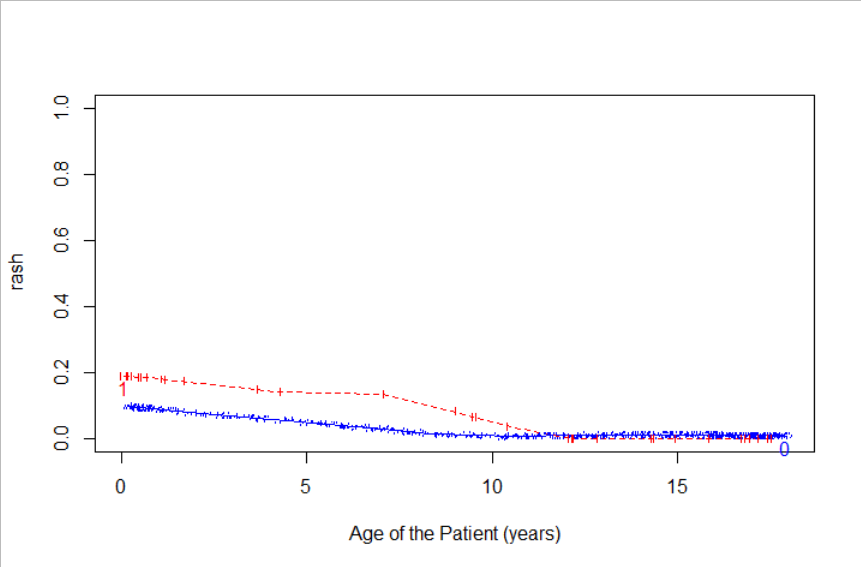


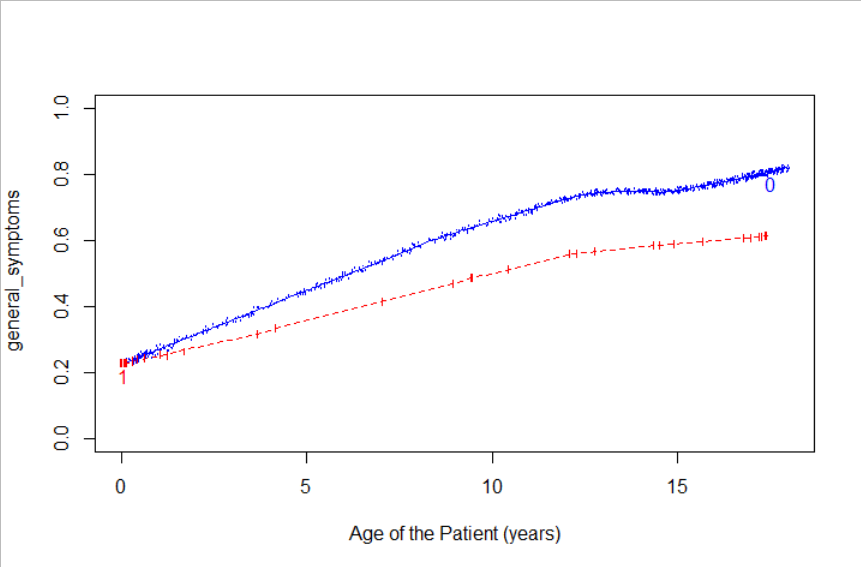

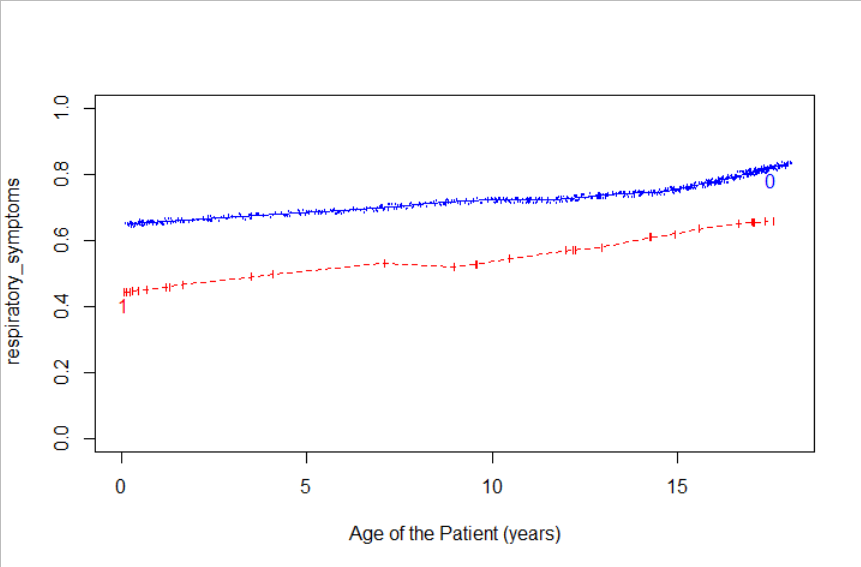

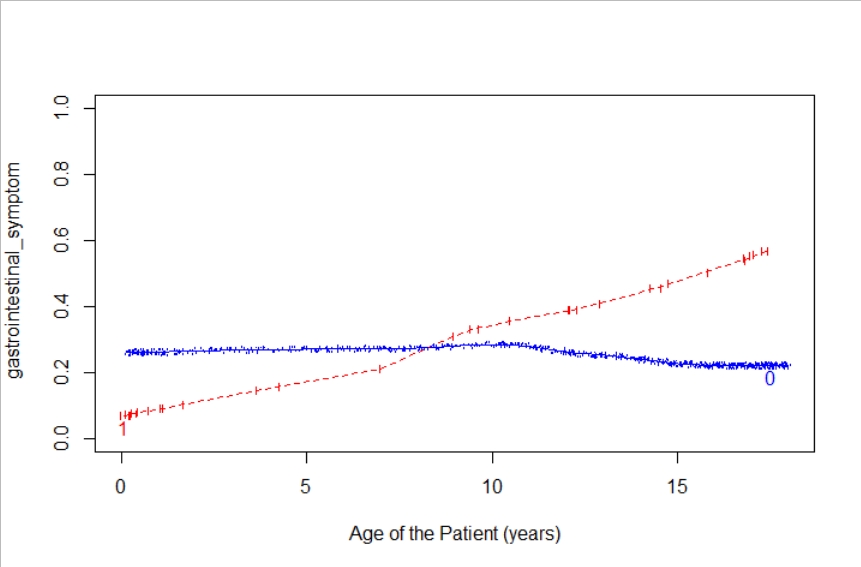


**Supplementary Table 2**. Clinical characteristics of hospitalizations among the first 1000 symptomatic pediatric SARS-CoV-2 infections in our network (n=41 patients hospitalized)

| Age group (yrs)^+^ | Any pre-existing condition | Immunocompromised | Neurologic condition | Cardiovascular condition | Chronic renal condition | Chronic lung condition | Diabetes | Other pre-existing condition | Indication for hospitalization | LOS^+^ (d) | COVID therapy |
| --- | --- | --- | --- | --- | --- | --- | --- | --- | --- | --- | --- |
| <1 |  |  |  |  |  |  |  |  | Febrile neonate | <2 | None |
| <1 |  |  |  |  |  |  |  |  | Febrile neonate | 2 | None |
| <1 |  |  |  |  |  |  |  |  | Brief resolved unexplained event | <2 | None |
| <1 | Yes |  |  |  |  |  |  | Yes | Brief resolved unexplained event | <2 | None |
| <1 | Yes |  |  | Yes |  |  |  |  | Apneic episodes | <2 | None |
| 1-4 | Yes |  | Yes | Yes |  | Yes |  | Yes | 1. Respiratory distress, hypoxemia 2. Cyanotic episodes | 1. <2 2. <2 | (1) None  (2) None |
| 1-4 |  |  |  |  |  |  |  |  | Diffuse skin desquamation, suspected Staphylococcal scalded skin syndrome | 4 | None |
| 1-4 | Yes |  | Yes |  |  | Yes |  |  | Respiratory distress, hypoxemia | 5 | None |
| 10-14 | Yes |  |  | Yes |  | Yes |  |  | Acute chest syndrome | 7 | Remdesivir (without concurrent steroids) |
| 10-14 | Yes |  |  |  |  |  |  | Yes | Hepatitis, presumed viral, no alternative etiology identified. Resolved at 2 week follow-up. | <2 | None |
| 10-14 | Yes |  | Yes |  |  |  |  |  | Respiratory distress, hypothermia increased seizure frequency | <2 | None |
| 10-14 |  |  |  |  |  |  |  |  | Dyspnea, orthostatic tachycardia | <2 | None |
| 10-14 | Yes | Yes |  |  |  |  |  |  | Fever and neutropenia | 12 | None |
| 10-14 | Yes |  |  |  |  |  |  | Yes | Pain crisis | 2 | None |
| ≥15 | Yes |  | Yes |  |  | Yes |  |  | Respiratory distress, hypoxemia | 11 | None |
| ≥15 | Yes |  | Yes |  |  |  |  |  | Feeding intolerance, dehydration, fever | 6 | None |
| ≥15 | Yes |  |  |  |  |  |  | Yes | Pain crisis | 8 | None |
| ≥15 |  |  |  |  |  |  |  |  | Hepatic abscess (culture from drainage procedure grew *Streptococcus constellatus*) | 6 | None |
| 5-9 | Yes | Yes |  |  |  |  |  | Yes | Chemotherapy | 5 | None |
| <1 |  |  |  |  |  |  |  |  | Subarachnoid hemorrhage after trauma | <2 | None |
| 5-9 |  |  |  |  |  |  |  |  | MIS-C | 7 | IVIG, steroids |
| ≥15 |  |  |  |  |  |  |  |  | Depression/suicidal ideation | 2 | None |
| ≥15 |  |  |  |  |  |  |  |  | Depression/suicidal ideation | <2 | None |
| ≥15 | Yes |  | Yes |  |  | Yes |  |  | Respiratory failure, pneumonia | 26 | Remdesivir and dexamethasone |
| <1 |  |  |  |  |  |  |  |  | Brief resolved unexplained event, fever | <2 | None |
| ≥15 | Yes |  |  |  |  |  |  | Yes | Fever, hypoxia, headaches, nausea, vomiting | 6 | Remdesivir and dexamethasone |
| 1-4 |  |  |  |  |  |  |  |  | Dehydration, dysphagia. Concurrent HSV gingivostomatitis | 4 | None; acyclovir for HSV |
| ≥15 | Yes |  |  |  | Yes |  |  |  | Pneumonia and shock | 14 | Remdesivir, dexamethasone, IVIG |
| ≥15 | Yes |  |  | Yes |  |  |  |  | Pain crisis | 11 | None |
| 5-9 | Yes |  | Yes |  |  |  |  |  | Fever and respiratory distress | 4 | None |
| 10-14 | Yes |  | Yes | Yes |  |  |  | Yes | Vomiting, dehydration, small bowel obstruction | 18 | None |
| ≥15 | Yes |  | Yes |  |  |  |  | Yes | Hypoxemia | <2 | Remdesivir (1 dose; stopped early due to rapid improvement) |
| <1 | Yes |  |  |  |  | Yes |  | Yes | Fever, hypoxemia | <2 | None |
| ≥15 | Yes |  |  |  |  |  |  | Yes | Emesis, dehydration | <2 | None |
| ≥15 | Yes | Yes |  |  |  | Yes |  |  | Cough, fever | 15 | Remdesivir, dexamethasone, IVIG |
| 5-9 | Yes |  |  |  | Yes |  |  |  | Pyelonephritis | 4 | None |
| 1-4 |  |  |  |  |  |  |  |  | Fever, vomiting, dehydration | <2 | None |
| <1 |  |  |  |  |  |  |  |  | Febrile neonate | 3 | None |
| ≥15 | Yes | Yes |  | Yes |  |  |  |  | Syncope, dyspnea | 6 | Remdesivir, prednisone |
| <1 | Yes |  |  | Yes |  |  |  |  | Hypoxia | 14 | None |
| ≥15 | Yes | Yes |  | Yes | Yes | Yes | Yes |  | Respiratory Failure | 52 | Dexamethasone, convalescent plasma |
| *+ Age in weeks (w), months (m), years (y); Length of stay (LOS) in days (d)* | | | | | | | | | | | |

**Supplementary Table 3. Predictive Model of Hospitalization.**

|  |  |  |  |  |
| --- | --- | --- | --- | --- |
|  | Non-hospitalized  (n=959) | Hospitalized  (n=41) | Unadjusted OR (95% CI) | P value |
| **Univariate** |  |  |  |  |
| Pre-existing conditions | 150 (15.6%) | 27 (65.9%) | 10.4 (5.3-20.3) | <0.001^d^ |
| Dyspnea | 81 (8.4%) | 17 (41.5%) | 7.7 (4.0-14.9) | <0.001^d^ |
| Black or Hispanic | 304 (31.7%) | 25 (61.0%) | 3.4 (1.8-6.4) | <0.001^d^ |
| Vomiting | 12 (1.3%) | 4 (9.8%) | 8.5 (2.6-27.7) | <0.001^d^ |
|  |  |  |  |  |
|  | Beta | Wald | Adjusted OR (95% CI) | P value |
| **Multivariate** |  |  |  |  |
| Pre-existing conditions | 2.046 | 32.5 | 7.7 (3.9-16.0) | <0.001^e^ |
| Dyspnea | 1.909 | 26.1 | 6.8 (3.2-14.1) | <0.001^e^ |
| Black or Hispanic | 1.684 | 7.6 | 2.7 (1.3-5.5) | <0.006^e^ |
| Vomiting | 1.684 | 5.7 | 5.4 (1.2-20.6) | <0.017^e^ |
| Constant/intercept | -4.884 | 182.1 | 0.008 | <0.001^e^ |
|  |  |  |  |  |
| d – denotes a P value based on a univariate logistic regression model with just the single predictor.  e – denotes a P value based on a multivariate logistic regression model with the 4 predictors.  AUC = 0.823 (95% CI 0.747-0.900) | | | | |

**Supplementary Figure 1**. Histogram presenting age distribution (years) among 1000 symptomatic SARS-CoV-2 positive children and adolescents diagnosed between 3/12/2020 and 9/28/2020

**Supplementary Figure 2**. Tree diagram of the factors in the model for hospitalization.

Legend. This classification and regression tree illustrated how the risk factors are related to hospitalization. Among the 1000 children with COVID-19, 4.1% (41) were hospitalized (1), 95.9% were not hospitalized. The strongest predictor of hospitalization was presence of a pre-existing condition (PEC). Among the 177 patients with a PEC, 15.3% were hospitalized vs. 1.7% of those without a PEC. Among those with a PEC, the next best predictor of hospitalization was shortness of breath (SOB). The hospitalization rate was 53.5% among those with both a PEC and SOB vs. 8.1% among those with a PEC but no SOB. Patients who are black or Hispanic were at increased risk of hospitalization among both those with and without PEC.


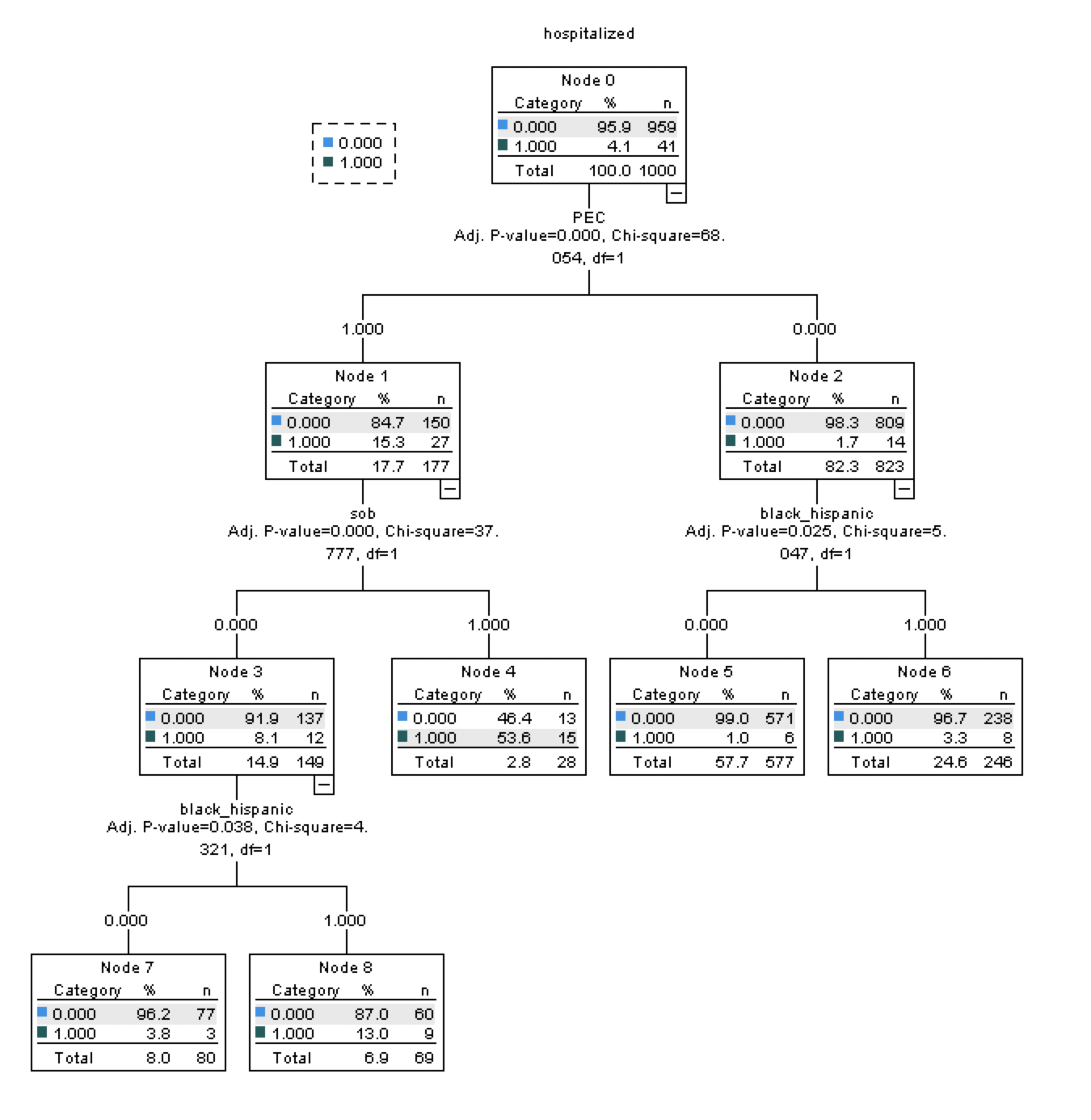


**Supplementary Figure 3**. Tree diagram of ‘pre-existing conditions’ in the model for hospitalization, and the relative contributions of immunologic, neurologic, and renal conditions to the predictive model.

**
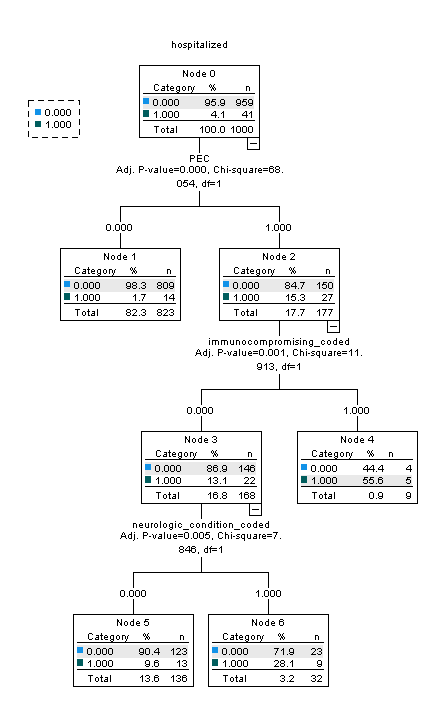
**

**Supplementary Figure 4.** Association between Age, Weight, and Hospitalization

Legend. The locally weighted scatter plot smoothing lines illustrate the relationship between weight and age in the hospitalization and non-hospitalized patients. The dark bands represent the 95% confidence interval of each line.


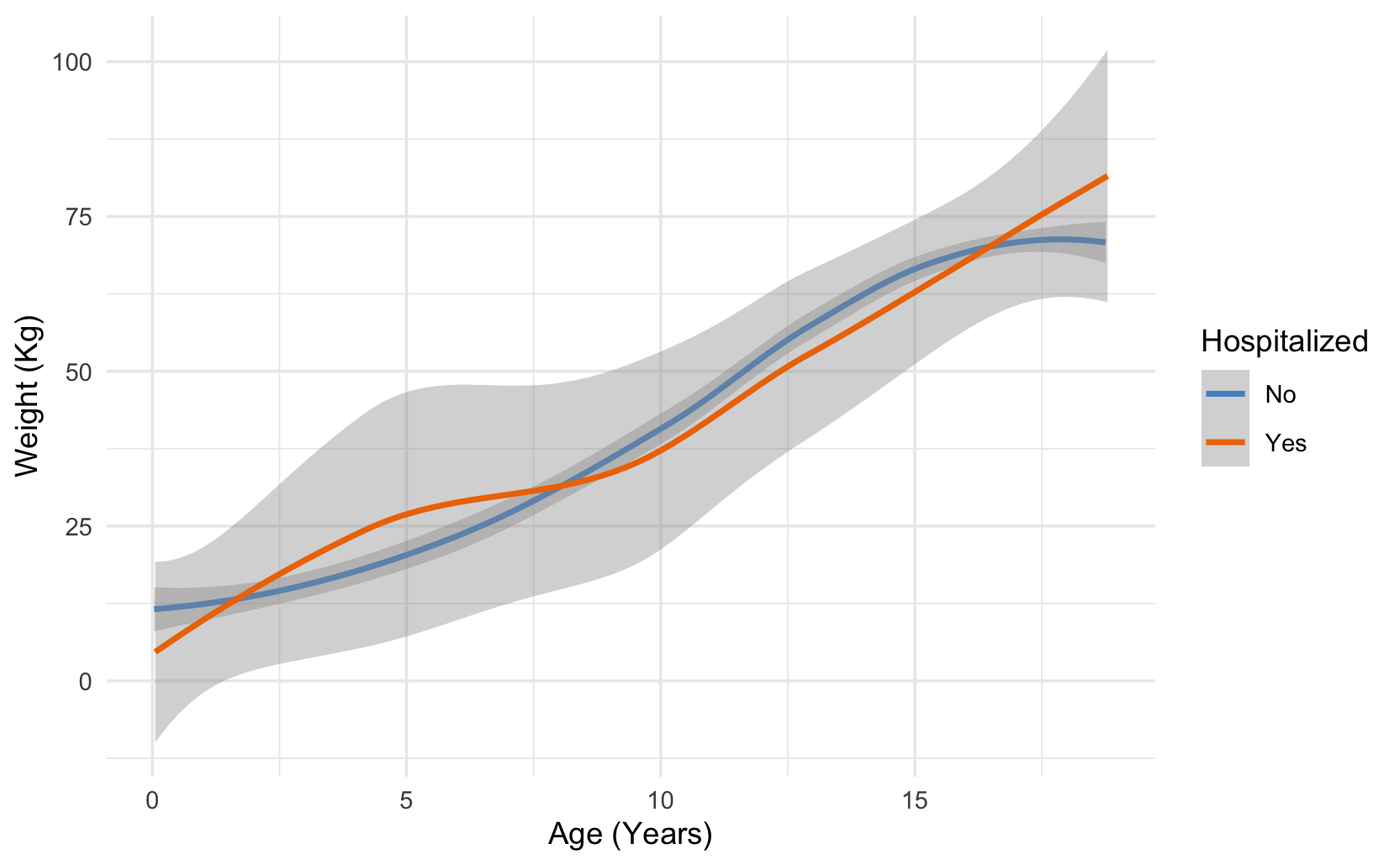


**Supplementary Figure 5. Calibration curve for multivariate model predicting hospitalization.**

Calibration plot showing predicted probability of hospitalization vs. observed rate of hospitalization.

**
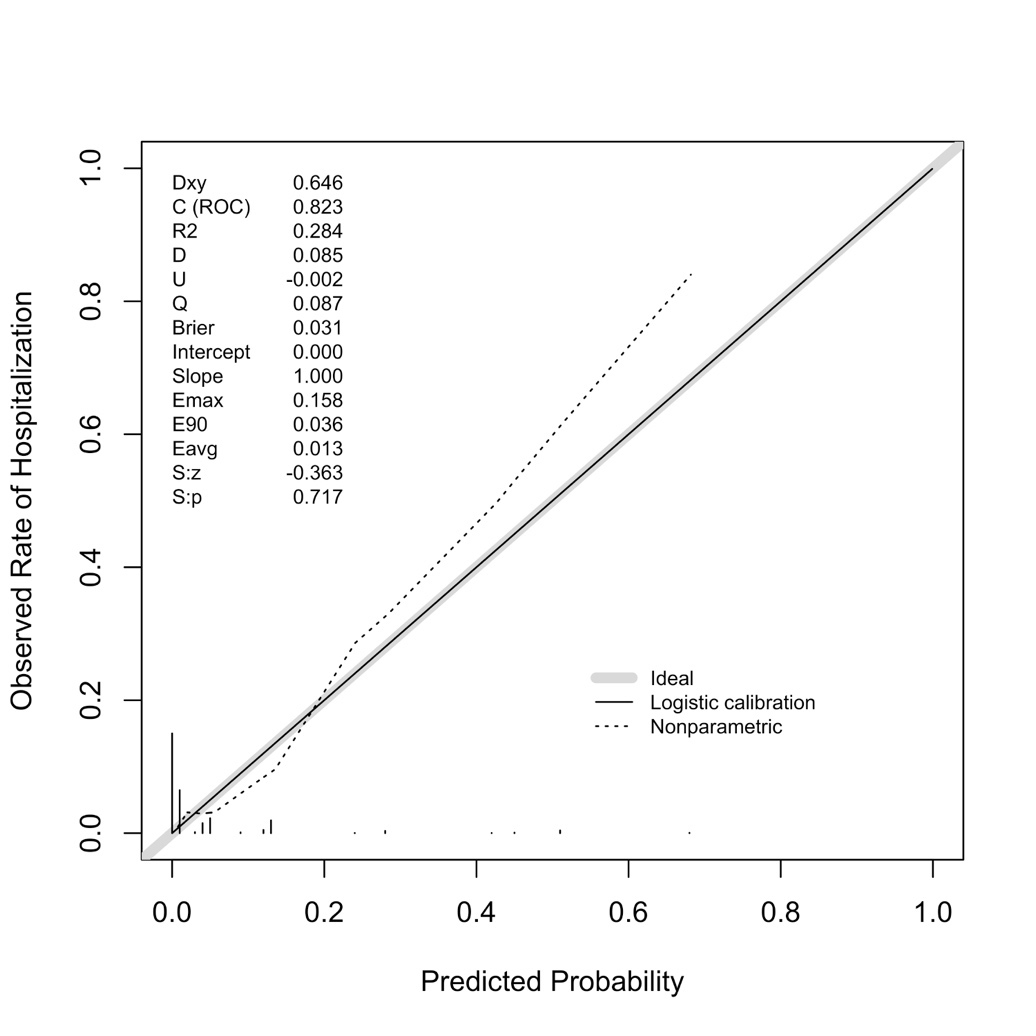
**
